# Congenital Esophageal Atresia Long-Term Follow-Up—The Pediatric Surgeon’s Duty to Focus on Quality of Life

**DOI:** 10.3390/children9030331

**Published:** 2022-03-01

**Authors:** Carlotta Ardenghi, Elettra Vestri, Sara Costanzo, Giulia Lanfranchi, Maurizio Vertemati, Francesca Destro, Ugo Maria Pierucci, Valeria Calcaterra, Gloria Pelizzo

**Affiliations:** 1 Pediatric Surgery Department, “Vittore Buzzi” Children’s Hospital, 20154 Milan, Italy; carlotta.ardenghi8@gmail.com (C.A.); elettra.vestri@asst-fbf-sacco.it (E.V.); sara.costanzo@asst-fbf-sacco.it (S.C.); giulia.lanfranchi@asst-fbf-sacco.it (G.L.); francesca.destro@asst-fbf-sacco.it (F.D.); ugomariapierucci@icloud.com (U.M.P.); 2 CIMaINa (Interdisciplinary Centre for Nanostructured Materials and Interfaces), University of Milano, 20133 Milan, Italy; maurizio.vertemati@unimi.it; 3 Pediatric Department, Children’s Hospital “Vittore Buzzi”, 20154 Milan, Italy; valeria.calcaterra@unipv.it; 4 Pediatrics and Adolescentology Unit, Department of Internal Medicine, University of Pavia, 27100 Pavia, Italy; 5 Department of Biomedical and Clinical Science “Luigi Sacco”, University of Milano, 20157 Milan, Italy

**Keywords:** esophageal atresia, children, congenital, pediatric surgery, quality of life, outcome

## Abstract

Esophageal atresia (EA) is the most common congenital esophageal malformation. An improvement in survival led to a focus on functional outcomes and quality of life (QoL). We analyzed the long-term outcomes and QoL of patients submitted to surgery for EA. Perinatal characteristics, surgical procedures, gastrointestinal and respiratory current symptoms and QoL were investigated. Thirty-nine patients were included. Long Gap patients had a higher rate of prematurity and low birth weight. The prevalent surgical procedure was primary esophageal anastomosis, followed by gastric pull-up. Twenty-four patients had post-operative stenosis, while gastroesophageal reflux (GER) required fundoplication in eleven cases. Auxological parameters were lower in Long Gap patients. The lowest scores of QoL were in the Long Gap group, especially in younger patients, which was the group with the highest number of symptoms. In the long term, the QoL appeared to be more dependent on the type of esophageal atresia rather than on associated malformations. Surgical management of GER was indicated in all patients with Long Gap EA, supposedly due to the prevalence of gastric pull-up for this type of EA. The assessment of QoL is part of surgeon’s management and needs to be performed in each phase of a child’s development.

## 1. Introduction

Esophageal atresia (EA) is the most common congenital esophageal malformation, with a prevalence of 1 in 3000–4000 [1,2,3,4]. In the last 20 years, there has been a large improvement in the associated survival; nowadays, EA has a survival rate greater than 90% [2,5,6], thanks to improvements in intensive care, neonatal anesthesia, ventilatory and nutritional support, antibiotic treatments and surgical techniques.

Despite significant improvements in the care of patients with EA, the management of complex esophageal atresia, largely represented by Long Gap forms, remains a challenge.

In the EA spectrum, Long Gap esophageal atresia (LG-EA) only accounts for a small percentage (10%), and it is defined as any EA in which there is inability to perform a primary esophageal anastomosis [7].

Preserving the native esophagus is the best option for patients [8]. However, both delayed anastomosis and esophageal replacement are associated with a high rate of postoperative complications, such as anastomotic stenosis and leak [9], dysphagia, airway infections, growth retardation and above all, gastroesophageal reflux disease (GERD) [10]. In fact, in all patients with EA, esophageal motility is impaired by denervation due to the surgical insult of the anastomosis [11,12]; moreover, in LG-EA, there is a shorter esophagus. Thus, after surgery, the gastro-esophageal junction is displaced, and the angle of His is distended. In the end, there is not a perfect substitute for the esophagus, and these patients frequently require more than one surgery, with an increased risk of surgical complications at each procedure, such as thoracic deformities.

With the reduction in mortality, the focus on functional outcomes and long-term quality of life (QoL) has increased [13,14]. Different aspects of life can be influenced by the long-term complications of this pathology, ranging from gastrointestinal to respiratory function, from nutrition to social activity and relation, therefore affecting overall QoL. Quality of life in EA patients has gained more and more importance over the years; QoL can be a valid indicator of how much the congenital pathology is going to affect patients in the long-term follow-up [13,14,15].

The goal of our study was to review long-term complications and the impairment of QoL during the development of patients submitted to surgery for EA.

## 2. Materials and Methods

### 2.1. Patients

We conducted a retrospective analysis of children who underwent surgery for esophageal atresia in the Pediatric Surgery Unit of Children Hospital V. Buzzi, born between 2004 and 2018, and with at least 3 years of follow-up.

Medical records were reviewed for personal and perinatal data, type of EA, associated anomalies, surgical treatment and complications and outpatient clinic annual anthropometric data follow-up. Gastrointestinal and respiratory current symptoms and QoL were also investigated through an online questionnaire collected by “Google Form”.

The follow-up included visits every 3 months for the first year of life, every 6 months in the following two years, then annual visits for the following years until adolescence, with the possibility to anticipate on the basis of patients’ symptoms.

All the parents or guardians consented to the retrospective management of anonymous data before the beginning of the survey in order to be enrolled in the study for clinical research purposes, epidemiology, the study of pathologies with the aim of improving knowledge, care and prevention [16]. Data were evaluated according to the principles of the Declaration of Helsinki, as revised in 2008. The privacy of the collected information was ensured according to Regulation (EU)/2016/679 GDPR, Legislative Decree n.101/18 and to General Authorization to Process Personal Data for Scientific Research Purposes (Authorization no. 9/2014).

### 2.2. Perinatal Data

Gestational age and birth weight were analyzed. Preterm birth is defined as any birth before 37 completed weeks of gestation. On the basis of gestational age and birth weight, the children were defined appropriate for gestational age (AGA) with a birth weight ≥10th percentile, small for gestational age (SGA) with a birth weight <10th percentile and large for gestational age (LGA) with a birth weight >90th percentile. Signs of EA in prenatal diagnosis were also included.

### 2.3. Type of EA and Associated Anomalies

The type of EA was defined according to the Gross classification [17]. The presence of one or more malformations including vertebral, cardiovascular, anorectal, renal, limbs or other pathologies were considered.

### 2.4. Surgical Information

Information about type of surgery was considered, such as primary or delayed anastomosis, age at the onset of anastomosis, gastric pull-up, gastrostomy placement, esophageal pouch tractions, second thoracotomy for anastomotic dehiscence, numbers of dilations to treat the stenosis and fundoplication surgery. Data about post-surgical complication were also analyzed, including stenosis or dehiscence of the anastomosis, the recurrence of tracheoesophageal fistula and the diagnosis of GER.

### 2.5. Anthropometric Data

The questionnaire included items about children’s current weight (kg) and height (cm). BMI was calculated by dividing body weight in kilograms by height in meters squared. Using an online calculator [18], percentiles of weight, height and BMI were collected, according to CDC growth charts [19].

### 2.6. Symptoms and Questionnaire of QoL

A questionnaire about QoL was submitted to all participants through an online document collected by “Google Form”, also investigating gastrointestinal and respiratory symptoms.

The symptoms included in the questionnaire were divided into two categories:

- Gastrointestinal symptoms such as heartburn, regurgitation, dysphagia, chest pain, nausea, vomiting during meals and diarrhea;
- Respiratory symptoms such as dyspnea, wheezing, cold, cough, night cough, choking, asthma and respiratory infections.

For every symptom, the frequency in the last year was asked, with 5 options and a relative score, where 0 was never, 1 was at least once a year, 2 was monthly, 3 was weekly and 4 was daily.

The quality of life was investigated by using a validated questionnaire reported by Dellenmark-Blom et al. (2017) [20]. The interview was divided into two groups of age:

- EA-QoL version for children 2–7 years old, parent-report. It included 17 items divided into 3 categories: “Eating”, “Physical Health and Treatment”, “Social Isolation and Stress”;
- EA-QoL version for children 8-17 years old, filled in by the patients themselves. It included 24 items, divided into: “Eating”, “Social Relationships”, “Body Perception”, “Physical Health and Well-Being”.

Each item had a score based on a 5-point Likert Scale, where higher points represented better quality of life. The final scores were linearly transformed to a 0 to 100 scale.

### 2.7. Statistical Analysis

All quantitative data were summarized as means and standard deviations (SDs) or medians and 95% confidence intervals as appropriate. Qualitative variables were converted into numerical ones and percentages. For the comparison between the populations, the analysis of variance test (ANOVA) was used, preceded by Levene and Welch statistics. Data were analyzed using SPSS Statistics. The significance level was *p* < 0.05.

## 3. Results

### 3.1. Clinical Features of Patients

A total of 57 patients met the chosen criteria. Thirty-nine of them answered the questionnaire, with a response rate of 68.4%. According to the Gross Classification, EA type C was present in 32 (82%) of patients, type A in 5 (12.8%), type B in 1 (2.6%) and type D in 1 (2.6%).

The mean age of the patients was 8.69 years, ranging from 3 to 16 years old. A time period of 8.69 years also corresponds to the average time of follow-up.

The characteristics of all patients, classified according to the presence of Long Gap, are reported in Table 1.

Prematurity was detected in 19 subjects (48.7%) and low birth weight in 19 patients (48.7%). A total of 25 patients (64.1%) had at least one associated anomaly, among which 16 were cardiovascular (41.03%), 6 were anorectal (15.38%), and VACTERL association was diagnosed in 4 of these cases (10.26%).

In patients with Long Gap, prematurity and low birth weight reached 83.33% (*p* = 0.003). The presence of at least one associated anomaly increased to 75% in patients with Long Gap, with a clear prevalence of cardiovascular anomalies (66.67%, *p* = 0.03), while for other categories, there was not a significant difference between the two samples (Table 1).

Ten patients (25%) had a prenatal diagnosis, with a statistically significant difference (*p* = 0.001) between the Long Gap sample (7 patients, 58.33%) and the Non-Long Gap sample (3 patients, 11.11%).

### 3.2. Surgery and Post-Surgical Complications

The main surgery for the correction of the esophageal defect, among all EA patients, was primary anastomosis (27 patients, 69.23%), followed by gastric pull-up (9 patients, 23.08%). The average gap at the first measurement in patients with LG-EA was 4.11 ± 1.36 vertebral bodies.

When needed, esophageal replacement surgery in LG-EA was performed with gastric pull-up (75%), while a delayed anastomosis was performed in the remaining 25%, in two cases after internal traction of the pouches. Almost all of the LG-EAs had a gastrostomy at birth.

Twenty-four post-operative stenosis (61.53%) were recorded during routine EGDS screening at 6 months and 1 year: eight were Long Gap (66.7%) and sixteen Non-Long Gap (59.3%). The number of dilations necessary to treat the stenosis in Non-Long Gap patients had an average of 2.26 ± 3.65, while in Long Gap, it reached 9.56 ± 13.41 (*p* = 0,047).

Gastroesophageal reflux required surgical correction with fundoplication in 11 patients (28.21%), 7 Long Gap and 4 Non-Long Gap patients. All the fundoplication surgeries were performed in children between 1 and 2.5 years old, with a mean age of 1.64 years.

### 3.3. Auxological Parameters

The average weight of the population was found at the 28.19 ± 24.60 percentile, average height at the 32.18 ± 29.28 percentile and average BMI at 35.29 ± 28.64 percentile. A lower percentile for weight, height and BMI was noted in Long Gap patients when compared with Non-Long Gap patients; however, this did not reach statistical significance (*p* = 0.06 for weight) (Table 2).

Patients with cardiac anomalies had a lower percentile in BMI (30th), weight (21st) and height (22nd) compared to the total population (Table S1). However, excluding patients with cardiac anomalies, the average percentiles remained lower than the average for normal population (for BMI, 39th, for weight, 33rd, for height, 39th).

### 3.4. Current Symptoms

**Figure 1:**
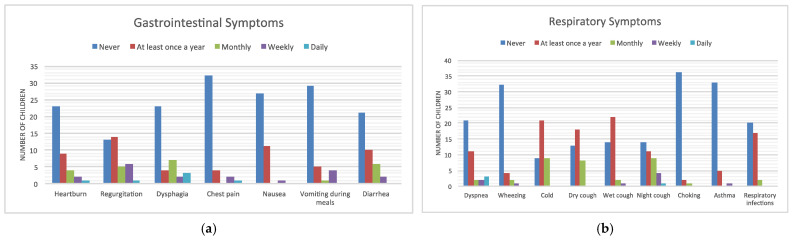
Prevalence and frequency of gastrointestinal (**a**) and respiratory symptoms (**b**).

The prevalence and frequency of symptoms is reported in Figure 1.

The most frequent gastrointestinal symptoms were regurgitation, with a mean score of 1.18, followed by dysphagia, diarrhea and heartburn, all with an average score of less than 1 (Table 3).

The largest number of patients indicated that they never suffered from the listed symptoms in the last year, except for regurgitation, for which 14 patients out of 39 (35.9%) indicated that they experienced it at least once a year. Dysphagia was not present in most children (23/39, 59%), although three patients reported it daily (Figure 1a).

Respiratory symptoms with an average score above 1 were cold and nocturnal cough, while other frequent symptoms were shortness of breath and dry cough (Table 4).

The symptoms experienced in the last year appeared to have higher percentages in the Long Gap sample than in the Non-Long Gap, both in the gastrointestinal and respiratory areas, but in both cases, statistically significant values were not achieved (Table 5).

### 3.5. Quality of Life

The average score of overall QoL in the population is 63.51%, with mean values of 68.06% among Non-Long Gap patients and of 53.27% among Long Gap patients, providing a statistically significant difference (*p* = 0.027).

In the questionnaire for the younger age group (2–7 years, *n* = 17), in all three categories, the scores were lower for the Long Gap sample. In both “Eating” and “Social Isolation and Stress” categories, the difference between Long and Non-Long Gap scores reached statistical significance (0.03). In the older age group questionnaire (8–17 years, *n* = 22), the difference was less marked; in fact, only in “Physical Health and Well-Being” category was there a statistical difference (*p* = 0.04) (Table 6).

We found no correlation between thoracic deformities and poor QoL or complications; however, in our population, there were no major deformities.

The anorectal malformations found in our patients were milder forms (including recto-perineal and recto-vestibular fistulas), with only one recto-urethral fistula. Therefore, the prognosis of these patients was good and there was not a significant influence on quality of life from a statistical point of view. The same stands for cardiovascular anomalies: there were mainly minor malformations (atrial septal defect, aberrant right subclavian artery…) and in only one case was a Tetralogy of Fallot recorded.

The median value between the total scores of the questionnaire was 66.18% and allowed us to create two subgroups, numerically similar (19 and 20 subjects), with higher and lower QoL. With that, we could compare these two QoL subgroups and their clinical characteristics, identifying possible elements impacting the QoL: the number of associated anomalies, preterm birth, low birth weight, gastrointestinal symptoms and respiratory symptoms. For all parameters, there was a numerical predominance in patients with lower QoL, but the only ones that reached significance were gastrointestinal symptoms (0.03) and respiratory symptoms (0.005) (Table 7).

A preliminary assessment was made comparing the average scores of questionnaires on QoL as well as the presence of gastrointestinal and respiratory symptoms, dividing the population according to gap length and to age group. The total scores of QoL were between 60 and 70% in all subgroups, except for the Long Gap EA in the younger age group, in which the score was only 41%.

Considering the number of both gastrointestinal and respiratory symptoms, there is a greater prevalence of gastrointestinal problems among younger patients with LG-EA. In fact, we found a score of 29% in this group of patients, while in the other subgroups (older patients and patients with Non-Long Gap EA), the values ranged from 14 to 17%.

## 4. Discussion

The study analyzed a population of patients with EA and reported long-term complications and impairment of QoL. Perinatal characteristics in these patients anticipate the outcome, which is less favorable in patients with LG-EA than in patients who had primary correction, and therefore, the need for chronic care in functional terms. However, the therapeutic path remains complex for Long Gap EA and exposes these patients to a greater risk of both short- and long-term complications, compared to other forms of EA.

The results of our study agree with the epidemiological studies reported in the literature, particularly with the Gross classification [17] which describes a prevalence of type C EA (85%) followed by type A (8%) [21]. Regarding perinatal characteristics and the comparison of patients with Long and Non-Long Gap EA, there is a significant difference in preterm births and low birth weight and in cardiological anomalies between the two groups. There is no relationship in the literature for congenital and perinatal characteristics and long-term outcomes; however, these are all factors known to contribute to higher surgical risks and increased morbidity in the post-operative stages.

Moreover, there is a statistically significant difference between the amount of Long Gap patients with prenatal diagnoses and the Non-Long Gap patients. This is in line with the literature, where a higher rate of polyhydramnios and prenatal diagnosis in EA without TEF is described [22].

From our series, up to 64.1% of patients had at least one associated anomaly, in line with the data from the EUROCAT register [2], which records a variable rate but greater than 50% of associated anomalies. The same applies to the proportion of patients with the VACTERL association, present in about 10% of the sample, as stated in the EUROCAT register [2]. Within the associated anomalies, only the cardiological ones reach a statistical difference between Long and Non-Long Gap groups, having an implication in the most commonly used prognostic classifications for EA according to the Spitz classification [23], which considers low birth weight and cardiac malformations as negative prognostic factors, and the more recent Okamoto classification [24], which focuses on major cardiac anomalies. These aggravating factors should be separated from the Long Gap condition to adequately evaluate the outcomes of this pathology. Surgery of LG-EA often requires delayed correction and the need of a gastrostomy at birth followed by several surgical steps before performing esophageal anastomosis. Each of these steps represent an increased predisposition to the development of surgical complications and a lower quality of life.

The main risk influenced by the gap length, according to the literature [9], seems to be the number of surgical complications and particularly the incidence of anastomotic dehiscence and stenosis.

In our patients, strictures were the most frequent complications (60% of the cases); this is in accordance with the literature, but frequently, the percentage described is around 40% [21,25]. Stenosis requiring dilations were shown to be more frequent in patients with LG-EA. The symptoms of the last year appeared to have higher percentages in the Long Gap sample than in the Non-Long Gap, but statistically significant values were not achieved.

The presence of dysphagia was reported, with a frequency ranging between every day and once a year, in 41% of children, with this result being lower than the one reported in the literature [26], where symptoms related to dysphagia are found in up to 50.3% of patients. Almost 46.15% of the children who completed the questionnaire had a current diagnosis of GERD, which reached 58.33% in the Long Gap population. This is in line with the literature [13], which indicates a prevalence of GER in patients with EA outcomes ranging from 22 to 56%, with higher rates in children with isolated EA. In our population, 28% required an anti-reflux procedure; according to the literature [21,27], anti-reflux procedures following EA are performed in approximately 30-40% of patients. The most interesting data are that the surgical management of GER was indicated in 100% of patients with Long Gap EA, while this was indicated in 30.8% patients with Non-Long Gap EA who had no response to medical treatment [28]. The high incidence of surgical treatment of GER in Long Gap EA could be influenced by the practice that in our unit, gastric pull-up is considered the technique of choice for the treatment of this type of EA. Gastric pull-up is described as the most used approach by many surgeons, probably as it is the simplest technique requiring a single anastomosis and having good vascularization of the stomach and an optimal position [7,29,30].

The complications are not increased compared to other techniques, except for the fact that the gastroesophageal junction is displaced, creating a hiatal hernia and distorting the angle of His, therefore increasing the incidence of GER [7,27]. These data are confirmed by the studies by Tovar et al. [31], in which a percentage close to 100% of fundoplication is found in patients with LG-EA, leading to the consideration that the Long Gap may be an indication for the surgical treatment of GER.

The high prevalence of growth failure [32] is related to surgical complications, associated anomalies, residual symptoms, comorbidities, prolonged hospitalization, as well as a history of GER and low birth weight. All these characteristics are more frequently found in Long Gap patients. In fact, growth also appears to be different between Long and Non-Long Gap patients, for all the evaluated parameters. In the comparison between the percentiles of weight, height and BMI, the average and the median always have higher values in the Non-Long Gap sample; these data can support the idea that in patients with Long Gap, there may be growth retardation. Additionally, in the entire population with EA, both Long and Non-Long Gap, the growth percentiles are always found in a lower range compared to the healthy pediatric population. Looking at the growth curves for BMI and weight, in each case, the fact that in the first years of life there is a reduction in growth in these patients is noticeable. A gradual recovery in growth was recorded during school age (5–10 years) followed by a reduction in growth curves from puberty to transitional age. Regarding the QoL, the lowest scores in the EA-QoL questionnaire appeared to be in the Long Gap group, especially in the younger patients, which is the same group of patients with the highest number of symptoms. This led us to hypothesize that patients who suffer the most in quality of life are the youngest, especially when the surgical management is complex. This is confirmed by other studies [32] in which the first years of life are considered the worst, with a higher complication rate and increased need of medical attention, while over 6–7 years of life, there is a gradual decrease in symptoms.

Among the parameters evaluated as potential precipitating factors of QoL, only the number of symptoms is significantly associated with score reduction, while the number of associated malformations, preterm birth and low birth weight, do not affect QoL in the long term. This underlines the fact that patients with a greater number of symptoms, rather than patients with associated congenital malformations, are those who also have a worse quality of life. Pediatric surgical follow-up must highlight the appearance of symptoms to adapt the management of the patient.

The number of symptoms is one of the factors that also impacts quality of life according to the authors of the questionnaire used in this study [15], together with delayed anastomosis, positioning of a gastrostomy, prematurity, esophageal dilations and associated anomalies.

As Flieder et al. [33] stated in 2019, our opinion is that, in the long term, the quality of life in these patients depends more on the type of esophageal atresia and thus on severity of the disease, rather than on the presence of associated malformations.

In the end, looking at the single categories included in the QoL questionnaire, an observation could be that in the “Eating” category, the worse scores are in younger patients, while the older ones develop coping skills, such as slowly eating or the need to increase drinking during meals, which decrease eating difficulties. They can adapt to the situation and manage their nutrition habits.

In the “Health” category, instead, there are worse scores in the older patients, indicating that they suffer more in terms of QoL, because of the respiratory difficulties under strain, in sport or playing activity, and because of the psychophysical sphere (difficulty sleeping, worrying about the future). In the respiratory field, an interesting fact is that many parents have pointed out that, with reduced participation in the community and the use of masks during the COVID-19 pandemic, respiratory infections were extremely reduced in number compared to the previous year [34]. This result underlines the fact that the occurrence of respiratory infections could also be strictly related to the environment.

Limitations of the study include the small sample size, which did not allow us to perform adequate statistical calculation, the lack of a standard value for the healthy population in the EA-QoL questionnaire, and the fact that it was a retrospective study; therefore, many data could not be collected. To overcome these limitations, a project for prospective and multicentric study could be an option.

## 5. Conclusions

The global care of EA patients emphasizes the importance of a long-term follow-up and the monitoring of the onset and the number of symptoms, which affect the QoL of children more than associated congenital malformations.

The assessment of QoL should be performed in each phase of a child’s development from infancy to adolescence and should be of priority importance in pediatric surgical follow-up. More data from long term outcome should be collected to allow pediatric surgeons to carry out tailored surgery to each type of esophageal malformation.

## Figures and Tables

**Table 1:** Clinical features of patients according to the presence of Long Gap.

	All (n = 39)			Long Gap (n = 12)			Non-Long Gap (n = 27)		
Variables	Absolute Number	Frequency (%)	Average ± SD/Median	Absolute Number	Frequency (%)	Average ± SD/Median	Absolute Number	Frequency (%)	Average ± SD/Median
Age (years)			8.69 ± 3.9/8			9.25 ± 4.11/10			8.44 ± 3.86/8
Male	26	66.67		7	58.33		19	70.37	
Prematurity	19	48.72		10	83.33 *		9	33.33	
Low birth weight	19	48.72		10	83.33 *		9	33.33	
Prenatal diagnosis	10	25.64		7	58.33 *		3	11.11	
At least one anomaly	25	64.10		9	75.00		16	59.26	
VACTERL	4	10.26		1	8.33		3	11.11	
Vertebral	3	7.69		0	0.00		3	11.11	
Cardiovascular	16	41.03		8	66.67 *		8	29.63	
Anorectal	6	15.38		2	16.67		4	14.81	
Renal	3	7.69		0	0.00		3	11.11	
Limbs	1	2.56		0	0.00		1	3.70	
Other	8	20.51		4	33.33		4	14.81	
Primary anastomosis	27	69.23		1	8.33 *		26	96.30	
Age at anastomosis (months)			2.49 ± 4.49/0			7.92 ± 4.78/6.5			0.07 ± 0.38/0
Gastric pull-up	9	23.08		9	75.00		0	0	
Gastrostomy *	14	35.90		11	91.67 *		3	11.11	
Surgical revision	4	10.26		1	8.33		3	11.11	
Dilations (No.) *			4.31 ± 8.18/1.5			9.56 ± 13.41/2 *			2.26 ± 3.65/1
Fundoplication	11	28.21		7	58.33		4	14.81	

* statistically significant difference between Long Gap and Non-Long Gap group.

**Table 2:** Average auxological parameters.

		Average	SD	Min	Max	*p*
Weight (Percentiles)	Non-Long	33.03	25.70	0.10	99.90	0.065
	Long	17.33	18.51	0.00	55.50	
	Total	28.19	24.60	0.00	99.90	
Height (Percentiles)	Non-Long	36.85	30.19	1.00	100.00	0.137
	Long	21.66	25.16	0.20	68.30	
	Total	32.18	29.28	0.20	100.00	
BMI (percentiles)	Non-Long	40.18	27.52	0.00	98.30	0.111
	Long	24.29	29.22	0.00	73.50	
	Total	35.29	28.64	0.00	98.30	

**Table 3:** Score of gastrointestinal symptoms.

	Heartburn	Regurgitation	Dysphagia	Chest Pain	Nausea	Vomiting	Diarrhea
Long Gap	0.92	1.50	1.00	0.42	0.33	0.17	0.50
Non-Long Gap	0.59	1.04	0.89	0.33	0.37	0.63	0.81
Total	0.69	1.18	0.92	0.36	0.36	0.49	0.72

**Table 4:** Score of respiratory symptoms.

	Dyspnea	Wheezing	Cold	Dry Cough	Wet Cough	Night Cough	Choking	Asthma	Respiratory Infections
Long Gap	1.25	0.25	0.92	0.58	0.92	1.50	0.33	0.25	0.58
Non-Long Gap	0.67	0.30	1.04	1.00	0.67	1.00	0.00	0.19	0.52
Total	0.85	0.28	1.00	0.87	0.74	1.15	0.10	0.21	0.54

**Table 5:** Average score of symptoms according to the length of the gap.

		Average	SD	Min	Max	ANOVA Sig.
Gastrointestinal Symptoms	Non-Long	4.11	5.071	0	24	0.230
	Long	6.08	3.476	0	11	
	Total	4.72	4.685	0	24	
Respiratory Symptoms	Non-Long	5.26	4.015	0	17	0.232
	Long	6.83	2.949	1	14	
	Total	5.74	3.754	0	17	

**Table 6:** Score of quality of life according to age group.

		No.	Average	p
Eating (2–7 years)	Non-Long	12	0.7262	0.027
	Long	5	0.4214	
	Total	17	0.6366	
Health (2–7 years)	Non-Long	12	0.6076	0.083
	Long	5	0.4000	
	Total	17	0.5466	
Isolation (2–7 years)	Non-Long	12	0.7292	0.025
	Long	5	0.4125	
	Total	17	0.6360	
Eating (8–17 years)	Non-Long	15	0.5104	0.694
	Long	7	0.4688	
	Total	22	0.4972	
Health (8–17 years)	Non-Long	15	0.8125	0.044
	Long	7	0.6339	
	Total	22	0.7557	
Relationship (8–17 years)	Non-Long	15	0.7048	0.927
	Long	7	0.7143	
	Total	22	0.7078	
Perception (8–17 years)	Non-Long	15	0.7967	0.471
	Long	7	0.7143	
	Total	22	0.7705	

**Table 7:** Score of quality of life in relation to clinical characteristics.

		No.	Average	SD	Min	Max	*p*
Associated Anomalies	Low	19	1.11	0.937	0	3	0.289
	High	20	0.80	0.834	0	3	
	Total	39	0.95	0.887	0	3	
Prematurity	Low	19	0.53	0.513	0	1	0.644
	High	20	0.45	0.510	0	1	
	Total	39	0.49	0.506	0	1	
Low Birth Weight	Low	19	0.53	0.513	0	1	0.644
	High	20	0.45	0.510	0	1	
	Total	39	0.49	0.506	0	1	
Gastrointestinal Symptoms	Low	19	6.37	5.776	0	24	0.030
	High	20	3.15	2.641	0	8	
	Total	39	4.72	4.685	0	24	
Respiratory Symptoms	Low	19	7.42	3.948	0	17	0.005
	High	20	4.15	2.815	0	11	
	Total	39	5.74	3.754	0	17	

## Data Availability

The data presented in this study are available on request from the corresponding author.

## Supplementary Materials

The following supporting information can be downloaded at: https://www.mdpi.com/article/10.3390/children9030331/s1, Table S1: Auxological data in patients with and without cardiac anomalies.
