# Supplementary material for: Congenital Esophageal Atresia Long-Term Follow-Up—The Pediatric Surgeon’s Duty to Focus on Quality of Life
Source: Children (Basel). 2022 Mar 1;9(3):331. doi: 10.3390/children9030331 (PMC8947008; doi:10.3390/children9030331)
Supplement: Supplementary file 1 [file children-09-00331-s001.zip › children-1599498-supplementary.pdf]

Supplementary Materials

Table S1. Auxological data in patients with and without cardiac anomalies.

Patients:	Without Cardiac Anomalies	With Cardiac Anomalies	Total
Average Weight (percentiles)	33.46	20.63	28.19
Average Height (percentiles)	39.24	22.02	32.18
Average BMI (percentiles)	38.94	30.04	35.29
